# Colonic lipoma mimicking malignancy and presenting as an intussusception: A rare case report

**DOI:** 10.1016/j.ijscr.2023.108611

**Published:** 2023-08-09

**Authors:** Shehryar Ahmed Khan Niazi, Muhammad Salar Raza, Muhammad Umer Mukhtar, Rameez Hassan, Muhammad Umar Nasir

**Affiliations:** a District Headquarter Hospital, Bhakkar, Pakistan; b Shaikh Zayed Medical Complex, Lahore, Pakistan; c King Edward Medical University, Lahore, Pakistan

**Keywords:** Gastrointestinal lipomas, Intussusception, Surgery, Case report

## Abstract

**Introduction and importance:**

Lipomas of the gastrointestinal tract are a rare entity compared to the more common tumors of the gut, such as adenomatous polyps and carcinomas. They were first described by Bauer in 1757. Gastrointestinal lipomas can grow in all segments of the gut, with the highest frequency in the colon. In this case report, we present a rare case of gastrointestinal lipoma mimicking colonic malignancy and causing intussusception, necessitating emergent surgery. This paper highlights the potential diagnostic challenges and therapeutic interventions associated with GI lipomas.

**Case presentation:**

A 28-year-old female presented with symptoms of abdominal pain, weight loss, vomiting, and changes in bowel habits. Initially, she received a misdiagnosis of Irritable Bowel Syndrome. Subsequent investigations indicated the possibility of colonic malignancy. During the intra-operative biopsy, it was ultimately discovered that she had a colonic lipoma.

**Clinical discussion:**

CT revealed an abdominal mass and an intussusception, indicating the need for surgical intervention. A laparoscopic procedure was performed to remove the mass, which alleviated the symptoms. Subsequently, a histological examination confirmed the mass to be a lipoma.

**Conclusion:**

Differentiating a gastrointestinal lipoma from malignancy is crucial, and careful investigation is necessary to determine if a local excision can be performed instead of a wide excision.

## Highlights

• Lipomas are benign tumors of the gastrointestinal tract that are most commonly found in the colon.
• Lipomas are typically asymptomatic, but they can cause symptoms such as abdominal pain, weight loss, and intussusception.
• Intussusception is a condition in which a portion of the intestine folds in on itself, which can cause blockage and pain.
• Keen clinical acumen and assessment allows pre-operative diagnosis and local resection instead of extended colectomy

## 1 Introduction

Lipomas are the second most common benign neoplasms of the colon after adenomatous polyps [1]. The reported incidence of lipomas relative to all polypoid lesions of the large intestine ranges from 0.035 % to 4.4 %, out of which only 17 % lead to colo-colic intussusception [2,3]. Most gastrointestinal tract lipomas are asymptomatic and are typically discovered incidentally. However, they can cause a wide range of non-specific symptoms or mimic malignancy. In 90 % of cases, they originate in the submucosa [4]. Lipomas can occur at any age, but they are most commonly found in individuals in their fifth and sixth decades of life, with a slight female predominance [5]. Before the availability of cross-sectional imaging, diagnosing lipomas was often challenging, as studies using digestive opacification could determine the benign nature of the tumor but not its specific cause. Currently, computed tomography (CT) and magnetic resonance imaging (MRI) are used to diagnose lipomas by revealing the pathognomonic greasy density or fat signal associated with these tumors. In this report, we present a rare case of a gastrointestinal lipoma that mimicked colonic malignancy and necessitated emergent surgery due to intussusception.

This case report has been reported following the SCARE guidelines [6].

## 2 Case presentation

### 2.1 History and exam

A twenty-eight-year-old woman presented to the emergency department with complaints of abdominal pain, postprandial vomiting, mucoid, tar-colored stools, and a weight loss of 18.6 kg over the past year. On abdominal examination a firm, non-tender mass with smooth margins measuring 3 × 2 cm was felt. Her symptoms initially began one year ago as intense, five-minute-long lower abdominal cramps after eating, accompanied by bloating. Over the next six months, her symptoms worsened to the point where she could only tolerate small liquid meals and required parenteral nutrition.

A past abdominal ultrasound had revealed a uterine myoma, leading to a myomectomy in January 2022. However, her initial symptoms persisted despite the surgery. This extended duration of symptoms, coupled with her complicated uterine myomectomy history, caused her significant anxiety. In March 2022, a sigmoidoscopy was conducted, which did not reveal any significant findings, although the exploration of the rest of the colon was hindered by poor gut preparation at the time. Despite seeking multiple consultations with various general practitioners, she was repeatedly misdiagnosed with irritable bowel syndrome and peptic ulcer. Laboratory tests showed a low hemoglobin level of 9 mg/dl. A subsequent endoscopy ruled out a peptic ulcer, and the antidepressants prescribed for irritable bowel syndrome failed to provide any relief.

### 2.2 Imaging and cytology

**Fig. 1:**
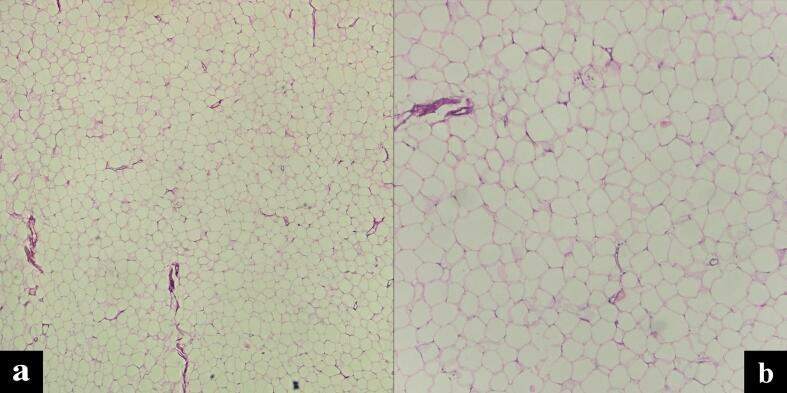
(a) and (b): Lobules of lipocytes with eccentric nuclei consistent with histology of lipoma (4×, 10×).

A contrast-enhanced CT scan was performed, which revealed a well-defined, round, concentric mass measuring 5.4 × 4.9 cm in the left hemiabdomen. Fat was observed in between the mass, along with a ‘bowel within bowel’ appearance (Fig. 1). The walls of the outer bowel loop and the central invaginated segment of the bowel showed normal enhancement. These findings were indicative of intussusception, most likely of colo-colic type. No free fluid was detected in the intussusceptum.

**Fig. 2:**
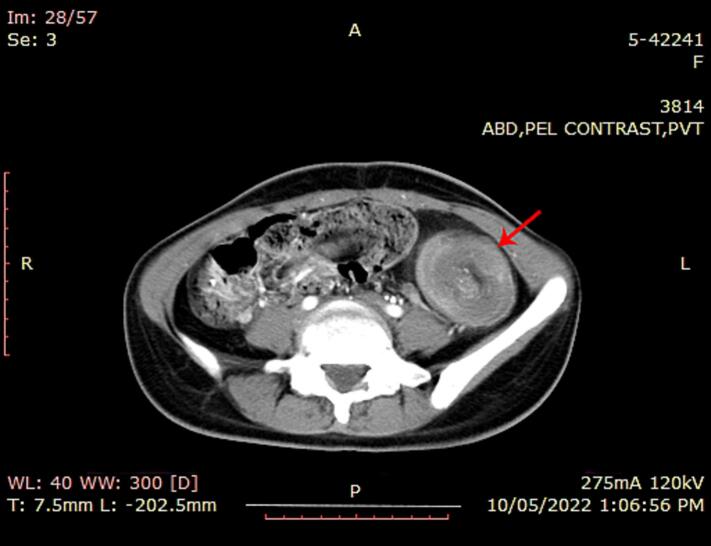
CT imaging with contrast showing concentric mass in left hemiabdomen with “bowel within bowel” appearance.

A biopsy was conducted via laparoscopic extended right hemicolectomy. Histological examination of the sections showed unremarkable intestinal mucosa, with mature adipocytes arranged in lobules separated by thin fibrous septa. No atypical cells or lipoblasts were observed, and there was no evidence of granuloma or malignancy (Fig. 2).

### 2.3 Treatment

**Fig. 3:**
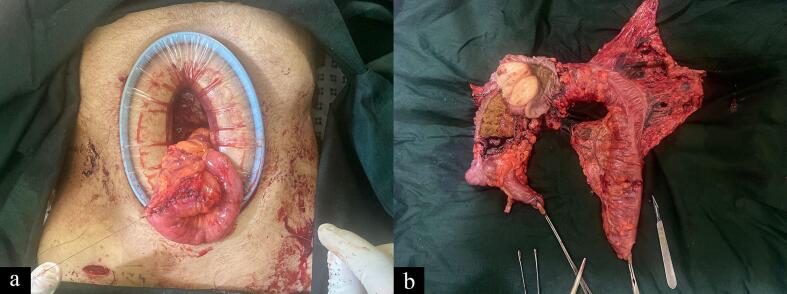
Intraoperative sections.

A definitive laparoscopic extended right hemicolectomy with ileocolic anastomosis was performed, during which the entire tumor was successfully removed (Fig. 3). The patient's postoperative recovery was optimal, and all of her complaints were resolved.

## 3 Discussion

Lipomas of the gastrointestinal tract are a rare condition and were first described by Bauer in 1757 [1]. Lipomas can occur anywhere in the intestinal tract, from the hypopharynx to the rectum, but they are mostly limited to one segment of the gut. The most common site is the ascending colon (45 %), while the rarest site is the transverse colon (9 %) [7]. The median age of patients with an intussuscepted colonic lipoma is forty-eight years, with a higher prevalence in females [4]. Only 25 % of GI lipomas exhibit clinical manifestations, which may include colonic intussusception [8,9]. If the tumor bleeds, patients may eventually develop anemia.

Intussusception is much more common in children than in adults. However, unlike children, where most cases are idiopathic, intussusception in adults has an identifiable etiology in 90 % of cases, with tumors being the most common lead points, as in our patient [10]. Depending on the position and size of the lipoma causing intussusception, symptoms may include abdominal pain, altered bowel movements, blood/mucus in the stool, vomiting, and partial or complete bowel obstruction. Symptomatic colonic lipomas continue to pose difficulties in the preoperative differential diagnosis between malignant and benign colonic neoplasms. Malignant colonic neoplasms and colonic lipomas may have similar symptoms, and both conditions may present with intussusception.

Regarding preoperative diagnostics, an abdominal CT scan is the preferred non-invasive radiological modality for colonic lipomas [8]. CT characteristics of lipomas include a spherical or ovoid shape, smooth and sharply demarcated margins, and thin fibrous septa. Pathognomonic imaging signs of lipomas include the “pillow mark” (a soft lesion with a cushion-like mucosal indentation when pressed with closed biopsy forceps) and the “bare fat mark” (leakage of fat after biopsy) [9]. Other investigation techniques, such as endoscopic ultrasonography and MRI, can also be very helpful [3,11].

Colonoscopy may allow direct visualization of the submucosal lipoma, and several characteristic endoscopic features have been described, including the “tenting sign” (grasping the overlying mucosa), “cushion sign” (flattening and restoration of the shape of the lipoma), and the “naked fat sign” (extrusion of fat after biopsy of the colonic mucosa) [12,13]. A colonoscopic biopsy is usually performed to confirm the nature of the tumor. However, inadequate tissue samples often indicate nonspecific colitis due to adjacent mucosal inflammation caused by lipoma.

Despite all these recent diagnostic innovations, it has been reported that the overall preoperative diagnostic accuracy is only about 62 %, and >70 % of patients require at least two instrumental investigations before receiving a diagnosis of intestinal intussusception caused by a lipoma. Our case illustrates the challenges that clinicians may face when diagnosing a GI lipoma [9].

Surgical intervention is necessary for treating symptomatic GI lipomas. Therapeutic interventions can range from segmental resection to local excision. The presence of intussusception is an indication of urgent surgery. In such acute situations, two treatment options are recommended, as discussed below:

1) If the colonic lipoma is diagnosed before intussusception, segmental resection is usually sufficient. Since more than half of intussusceptions occur within a single anatomical segment of the colon, most patients require varying degrees of colon resection based on the tumor's location and the length of the intussuscepted segment. Preoperative diagnosis assists surgeons in optimizing the surgical treatment for intussusception. Despite being a benign tumor, intraoperative frozen sections are necessary to ensure negative surgical margins and prevent a recurrence.

2) Two-thirds of colonic intussusceptions are caused by primary adenocarcinoma. Therefore, if a lipoma is not diagnosed before surgery, it must be treated as cancer, and an extensive section of the colon needs to be removed. In such cases, the histological diagnosis is determined only after the tumor has been excised.

As far as our patient was concerned, we performed an intraoperative frozen section, confirming tumor-free margins of 5 cm [14]. We then restored the ascending-transverse intussusception and performed an extended right hemicolectomy with ileocolic anastomosis.

The size of the lipoma is a crucial factor in colonic intussusception. Lipomas that cause intussusception typically range in size from 4 to 16 cm, with an average size of 7 cm. Several studies have shown that the removal of lipomas with a diameter ≥ 2 cm carries a higher risk of perforation [15]. Some authors have reported the successful removal of large pedunculated and sessile lesions without perforation using *endo*-clipping or endoloop ligation [16]. In our opinion, when a colonic lipoma is diagnosed with a size of 4 cm or more, surgical intervention is necessary to prevent intussusception. The most recent published data comparing laparoscopic and open colorectal resection for lipomas recommend laparoscopic resection as the preferred method for removing lipomas larger than 2 cm in diameter, even in cases where the tumor's malignant nature has not been ruled out preoperatively [4,17].

## 4 Conclusion

Symptomatic intestinal lipoma is a rare condition. It can imitate malignancy by inducing weight loss and serving as a lead point for intussusception. The diagnosis of intestinal lipoma necessitates a considerable degree of suspicion and the utilization of preoperative radiological and histopathological examinations. Treatment typically involves standard resection with wide free margins to minimize the chances of recurrence. This paper emphasizes the significance of a carefully established preoperative diagnosis to avoid excessive resection.

## CRediT authorship contribution statement

**Shehryar Ahmed Khan Niazi:** Conceptualization, Resources, Data curation, Writing- Original draft preparation, Writing - Review & Editing **Muhammad Salar Raza:** Conceptualization, Software, Writing- Original draft preparation, Writing - Review & Editing, Visualization. **Muhammad Umer Mukhtar:** Writing- Original draft preparation, Resources. **Rameez Hassan:** Project administration, Writing - Review & Editing. **Muhammad Umar Nasir:** Methodology, Investigation.

## Grant support & financial disclosures

None.

## Consent

Written informed consent was obtained from the patient for publication and any accompanying images. A copy of the written consent is available for review by the Editor-in-Chief of this journal on request.

## Source of funding

This research did not receive any specific grant from funding agencies in the public, commercial, or not-for-profit sectors.

## Ethical approval

Ethical approval was obtained from the head of the Department of Surgery, DHQ Bhakkar, Pakistan on 16 April 2023. The institute doesn't grant specific numbers to approvals granted to case reports.

## Registration of research studies

Name of the registry: UMIN-CTR.

Unique identifying number or registration ID: UMIN000051169.

Hyperlink to your specific registration (must be publicly accessible and will be checked): https://center6.umin.ac.jp/cgi-open-bin/ctr_e/ctr_view.cgi?recptno=R000058359

## Guarantor

Shehryar Ahmed Khan Niazi.

## Declaration of competing interest

The authors have no conflict of interest to declare.
